# Subcutaneous Extravasation of Sr-89: Usefulness of Bremsstrahlung Imaging in Confirming Sr-89 Extravasation and in the Decision Making for the Choice of Treatment Strategies for Local Radiation Injuries Caused by Sr-89 Extravasation

**Published:** 2013

**Authors:** Joji Kawabe, Shigeaki Higashiyama, Kohei Kotani, Atsushi Yoshida, Hiroyuki Tsushima, Takashi Yamanaga, Daisuke Tsuruta, Susumu Shiomi

**Affiliations:** 1 Department of Nuclear Medicine, Graduate School of Medicine, Osaka City University; 2 Department of Radiological Sciences, Ibaraki Prefectural University of Health Sciences; 3 Department of Radiology, Osaka City University Hospital; 4 Department of Dermatology, Graduate School of Medicine, Osaka City University

**Keywords:** Sr-89, Extravasation, Local radiation injury, Bremsstrahlung imaging

## Abstract

A male patient in his 20s presented at our clinic with pain caused by bone metastases of the primitive neuroectodermal tumor, and Sr-89 was administrated to palliate the pain. After receiving the injection, the patient complained of a slight burning pain at the catheterized area. Slight reddening and small circular swelling (diameter, 0.5 cm) were observed at the catheterized area. Sr-89 extravasation was suspected. To estimate the amount of subcutaneous Sr-89 leakage, bremsstrahlung imaging was immediately performed. We speculated that the skin-absorbed dose from subcutaneous infiltration of Sr-89 was 1.78 Gy. The mildest clinical sign of local radiation injury was erythema. The received dose was higher than 3 Gy, and the time of onset was from 2 to 3 weeks. In our patient, local radiation injuries (LRIs) did not occur. Though requiring further verification, subsequent bremsstrahlung imaging and estimation of the skin-absorbed dose from the subcutaneous infiltration of Sr-89 are useful in confirming Sr-89 extravasation and in the decision making for the choice of treatment strategies for LRIs caused by Sr-89 extravasation.

## Introduction

Reports on cases of local radiation injuries (LRIs) caused by the extravasation of therapeutic radionuclides are rare. In a PubMed search, a report on Y-90 extravasation was found (1), but none on Sr-89 extravasation. With the increasing use of therapeutic radionuclides, a corresponding increase in LRI cases resulting from possible extravasation is expected. When the extravasation of radionuclide is suspected at injection, extravasation should be confirmed and the radionuclide dose absorbed by the skin should be measured (2, 3). Here, we present a case of Sr-89 extravasation, for which we used bremsstrahlung imaging to identify and estimate the amount of subcutaneous Sr-89 leakage and predict the severity of a possible LRI.

## Case Report

A male patient in his 20s presented at our clinic with pain caused by bone metastases of the primitive neuroectodermal tumor, and Sr-89 was administrated to palliate the pain. The patient was intravenously catheterized at the right cubital vein, using a 21-G winged needle. After confirming retrograde blood flow in the vein, the patient was slowly injected with 136 MBq of chloride Sr-89 with 30-mL saline. After receiving the injection, the patient complained of a slight burning pain at the catheterized area. Slight reddening and small circular swelling (diameter, 0.5 cm) were observed at the catheterized area. Extravasation of the injected fluid was suspected; in accordance with the manual for the management of possible extravasation, (2) immediate warming and massaging of the catheterized area were started. To estimate the skin-absorbed dose of Sr-89 from the subcutaneous leakage, bremsstrahlung imaging was subsequently performed, using an ADAC Forte gamma camera fitted with a low-energy high-resolution (LEHR) collimator. The pulse-height analyzers were set at a 100-keV photopeak, with a window width of 25% and scan velocity of 15 mm/min. Images were acquired in a 1024×512 word mode matrix. Generally, bremsstrahlung imaging is performed 1 week after Sr-89 injection, using a medium-energy collimator (4-6). However, in our case, the images were obtained immediately after Sr-89 injection, using LEHR collimators. However, an anterior view of the whole-body scan could not be obtained on bremsstrahlung imaging because of the mistake of the photopeak setting of the anterior detector of the pulse-height analyzer.

**Figure 1:**
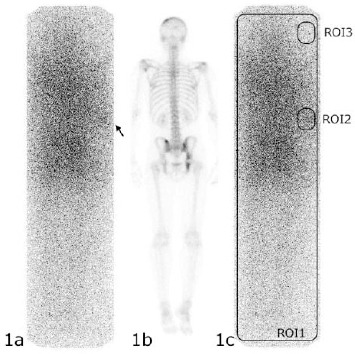
a. Posterior view of the whole-body scan on bremsstrahlung imaging.

Figure 1a shows the posterior view of the whole-body scan on bremsstrahlung imaging. Figure 1b depicts the posterior view on bone scintigraphy. As opposed to the bone scintigram, the bremsstrahlung scan showed an abnormal uptake (arrow) in the elbow area. The skin-absorbed dose of Sr-89 from the subcutaneous leakage was calculated using the method by Yamaguchi *et al* (3, 7, 8) in accordance with the manual for the management of possible extravasation (2). Regions of interests (ROIs) were determined based on whole-body uptake (ROI1; 34060 pixels), the abnormal uptake (ROI2; 492pixels) and the background uptake (ROI3; 492pixels) (Figure1c). Total counts of ROI1, ROI2 and ROI3 provided 63879, 1114 and 540 counts, respectively. Mean Counts of the three ROIs provided 1.875, 2.264 and 1.111, respectively.

The injected Sr-89 dose was 136 MBq, and the amount of subcutaneous Sr-89 leakage was estimated to be about 2.96 MBq (=136 MBq× { (492×2.264 - 492×1.111) / 34060×1.875 - 34060×1.111}}). It was presumed that the absorption time of Sr-89 into the blood through the skin was 30 minutes (effective half-life of retention in the site of injection, 180 seconds (2, 3, 8); the swollen area was approximately 0.2 cm^2^ (=0.25 cm×0.25 cm×3.14). In accordance with the formula of the manual (2, 7), the skin-absorbed dose from the subcutaneous Sr-89 leakage could be calculated by the following formula:

The skin-absorbed dose from the subcutaneous Sr-89 infiltration (mGy)=T/0.693×A/Area ×Dr While:

T=effective half-life (seconds) (2, 3, 8)

Dr=skin-absorbed dose ratio (8) (1667 nGyn-−1Gyn−1Gyn^2^)

A=subcutaneous leakage dose (MBq)
Area= contaminated area (cm^2^)
Using above formula in our patient:
Skin absored dose = {(180/3600)/0.693} × (2.96/0.2) × 1.667= 1.78 Gy

A dermatologist applied lotions containing hydrocortisone and covering to the injury site. On the follow-up checkup the next day, the redness and swelling in the injury site were no longer visible. Generally the mildest clinical sign of local radiation injury is erythema. The reported estimated skin dose is higher than 3 Gy, and the time of onset of symptoms is from 2 to 3 weeks after injection. (9) In our patient, we calculated the skin-absorbed dose from subcutaneous infiltration of Sr-89 and it was 1.78 Gy. Then, we predicted correctly that LRIs would not occur in our patient.

## Discussion

Since the first report (3) of LRI caused by subcutaneous Tl-201 extravasation in 2001, the methods of measuring skin-absorbed dose from the subcutaneous Sr-89 infiltration have been evaluated and improved, (3, 7, 10) leading to the development of safety manuals for the management of therapeutic radionuclide extravasation. (2, 11) Williams *et al* (1) indicated that morbidity of LRIs may be reduced by identifying extravasation at the intravenous injection site of the radionuclide and by providing early treatment immediately after confirming the extravasation.

Safety guidelines for suspected Sr-89 extravasations, have the following recommendations (1, 2, 10, 11):

1) Mark the region of extravasation (if possible, take a photograph of the region).
2) Perform warming to promote vasodilatation and possible removal and dilution of the extravasated Sr-89 and to relieve pain.
3) Consider administration of steroids.
4) As soon as possible, monitor the remaining fraction of the skin-absorbed dose from the subcutaneous Sr-89 infiltration by bremsstrahlung imaging.
5) Consult a dermatologist.

In the manual, performing bremsstrahlung imaging was not mentioned. Skin-absorbed dose of Sr-89 from subcutaneous leakage was calculated in accordance with the method of Yamaguchi *et al* (3, 7, 8) as described by Kobayashi *et al* (3). Yamaguchi *et al* assumed that the amount of radionuclide extravasation is approximately one-third of the whole quantity. In the manual (2, 3) the average ratio of Sr-89 extravasations was assumed to be 30%. However, the ratio varies according to each case. We actually measured the ratio by bremsstrahlung images. Yamaguchi *et al* supposed that the contaminated area of the Sr-89 from subcutaneous leakage was 10 cm^2^. According to the inspection, instead of 10 cm^2^, we supposed that the contaminated area was the small circular swelling (diameter, 0.5 cm) in the catheterized area in order not to underestimate the skin-absorbed dose from the subcutaneous Sr-89 infiltration. Other calculating methods of skin absorbed doses due to subcutaneous leakage of radioactive pharmaceuticals were proposed by Minsky *et al* (12), Shapiro *et al* (13) and ICRU Report 56 (8) and so on. Kobayashi *et al* illustrated that the calculating method by yamaguchi *et al* was the most suitable for the supposition of the skin absorbed doses.

In this case, the square ROI (ROI 1) and the background ROI (ROI 3) were determined instead of the ROI surrounding the whole body uptake, because it was difficult to determine along the counter of the whole body uptake correctly and in order to remove the influences from the environment background and scatter radiations. As a result, overestimation of uptake and absorbed dose may be seen. Determination of square ROI was very easy and objective. This measurement of the ratio of Sr-89 extravasation was thought to be clinically useful. More study maybe needed to accurately define ROI in these patients.

In our case, bremsstrahlung imaging was performed immediately after radionuclide injection, which had 2 benefits. First, it allowed us to confirm the occurrence of extravasation. Second, it enabled us to perform quantitative measurement of the ratio of Sr-89 extravasation. The measurement was clinically useful for the treatment strategy and the prediction of prognosis of this patient. The bremsstrahlung imaging technique that we used in the present case requires further technical evaluation. Oda *et al* (14) indicated that setting the energy window at 75 keV (window width of 50%) with the use of a medium energy low penetration collimator (MELP) collimator is optimal for imaging. Next time, the pulse-height analyzers may be set at 75 keV photo-peak with a window width of 50% with MELP collimator.

## Conclusion

When subcutaneous extravasation is suspected after injection of Sr-89, we would recommend performing bremsstrahlung imaging immediately and starting supportive treatment early.
